# Mitochondrial Compartmentalized Protein Folding and Tumor Cell Survival

**DOI:** 10.18632/oncotarget.273

**Published:** 2011-05-07

**Authors:** Dario C. Altieri

**Affiliations:** Prostate Cancer Discovery and Development Program, The Wistar Institute Cancer Center, Philadelphia, PA 19104

**Keywords:** Hsp90, mitochondria, chaperone, unfolded protein response, cancer therapy

## Abstract

Molecular chaperones are master regulators of protein folding quality control, and it is widely accepted that these functions are aberrantly exploited in human tumors. What has also emerged in recent years is that chaperone control of protein folding does not occur randomly in cells, but is spatially compartmentalized in subcellular organelles and specialized microenvironments. Fresh experimental evidence has now uncovered a role for mitochondrial localized chaperones to oversee the protein folding environment within the organelle, selectively in tumor cells. Perturbation of this compartmentalized chaperone network triggers an array of compensatory responses that aims at restoring homeostasis, while also providing novel opportunities for rational cancer therapy.

## INTRODUCTION

Built on a better understanding of cancer genes and their pathways [1], it is now possible to encompass cancer signaling pathways in their globality, as orchestrated, interconnected *networks*. Taking advantage of systems biology tools [2], this approach may be better suited to capture the complexity of tumor cells, and account for typical traits of malignant growth, such as heterogeneity, redundancy, and buffering [3]. The charting of network connectivity maps [2] may also uncover the function of so-called *nodal* proteins in tumors, hub molecules that control multiple downstream subnetworks, and whose therapeutic inhibition may provide desirable anticancer activity in genetically and molecularly heterogeneous tumors.

Molecular chaperones neatly fulfill the definition of cancer nodal proteins [4]. Fueled by the energy produced by ATP binding and hydrolysis, and assembled as supramolecular protein complexes, molecular chaperones, especially members of the Heat Shock Protein (Hsp) family, oversee the global process of protein folding quality control, impinging in virtually every aspect of cellular homeostasis [4]. We also know that chaperone control of protein folding does not occur randomly in cells, but is compartmentalized in subcellular organelles and specialized microenvironments. The list of these sites has grown considerably over the years, and now includes the endoplasmic reticulum (ER), mitochondria, nuclei, the plasma membrane and even the extracellular environment [4].

Now, recent evidence suggests that the mitochondrial pool of molecular chaperones [5] maintains the organelle protein folding environment in tumor cells, and is hard-wired to the transcriptional stress response machinery of cell adaptation and cell survival [6].

## MITOCHONDRIAL CHAPERONES IN THE CONTROL OF TUMOR CELL SURVIVAL

Results from our group recently uncovered a network of molecular chaperones that localize to mitochondria, almost exclusively in tumor cells [7]. For some of these molecules, for instance the Hsp90 homolog, TNF Receptor-Associated Protein-1 (TRAP-1) [5], and the chaperonin Hsp60 [8], a mitochondrial localization had been described before, and either linked to unknown functions or the refolding of proteins that cross the mitochondrial membrane, respectively. Instead, a mitochondrial pool of Hsp90 had not been previously described, anticipating a broader role of this chaperone in organelle homeostasis [5]. It is still unclear how Hsp90 accumulates preferentially in mitochondria of tumor cells, compared to normal tissues, but earlier work suggested a potential role of oncogenic signaling, but not metabolic unbalance, in this pathway [5].

Once in mitochondria, Hsp chaperones form a physical complex with cyclophilin D (CypD) [5], an immunophilin component of the organelle permeability transition pore, or at least a pivotal regulator of it [9]. A detailed structure-function relationship of CypD-chaperone complex(es) is still missing, but there is initial evidence that mitochondrial Hsp90, TRAP-1, and Hsp60 may simultaneously bind CypD through non-overlapping recognition sites. In turn, the multichaperone-CypD complex antagonizes the opening of the mitochondrial permeability transition pore, potentially by protein (re)folding, shutting off the initiation of apoptosis in tumors [7]. Mechanistically, this pathway appears ideally suited to globally elevate the anti-apoptotic threshold in transformed cells [7], favoring the acquisition of additional malignant traits, including adaptation to unfavorable, i.e. hypoxic, environments, and resistance to conventional or targeted therapy.

For this reason, mitochondrial Hsp chaperones provide attractive targets for cancer therapeutics. Embodying the concept of subcellularly-targeted therapy for human diseases, a novel class of small molecule Hsp90 inhibitors was recently engineered to target the chaperone pool selectively in mitochondria. When tested in preclinical models, these agents, called Gamitrinibs (geldanamycin –GA- mitochondrial matrix inhibitors) induced sudden collapse of organelle integrity, with apoptotic killing of heterogeneous tumor cell types [10], and inhibition of localized and metastatic tumor growth in mice. Because molecular chaperones are virtually absent, and anyway uncomplexed with CypD in normal mitochondria [8], Gamitrinib-based therapy was well tolerated, with no detectable toxicity for normal cells or tissues, in vivo [7].

## HARD-WIRING OF MITOCHONDRIAL CHAPERONES WITH THE CELLULAR STRESS RESPONSE IN TUMORS

**Figure 1:**
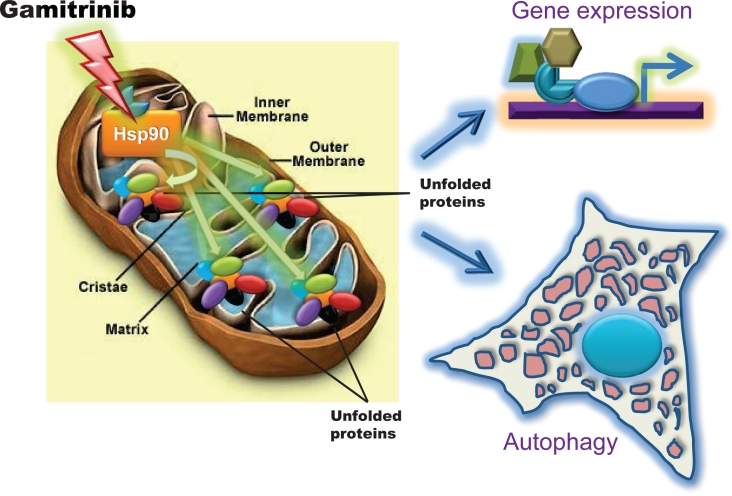
Bipartite mitochondrial UPR induced by targeting organelle Hsp chaperones Treatment of tumor cells with low, i.e. non-cytotoxic concentrations of mitochondrially-targeted Hsp90 inhibitor, Gamitrinib induces a proteotoxic response within accumulation of unfolded proteins within the organelle and resulting in activation of autophagy and a stress response gene expression signature.

Now, recent data have shown that Gamitrinib, especially the variant containing triphenylphosphonium as mitochondriotropic moiety [7], triggered an entirely new signaling pathway in tumor cells. Using glioblastoma as a model, it was recently shown that sub-optimal concentrations of Gamitrinib that have minimal or no effect on cell viability, dramatically activated autophagy, with extensive cytoplasmic vacuolation and lipidation of the autophagy marker, dynein light chain 3 [6] (Fig. 1). It is still debated whether autophagy is a form of cell death, a survival mechanism, or both [11]. In the case of Gamitrinib, this pathway was clearly a compensatory response aimed at maintaining cell viability, as pharmacologic inhibitors of phagosome formation or small interfering RNA (siRNA) knockdown of the essential autophagy gene, atg5, converted non-toxic concentrations of Gamitrinib into active doses that killed glioblastoma cells by apoptosis [6].

**Figure 2:**
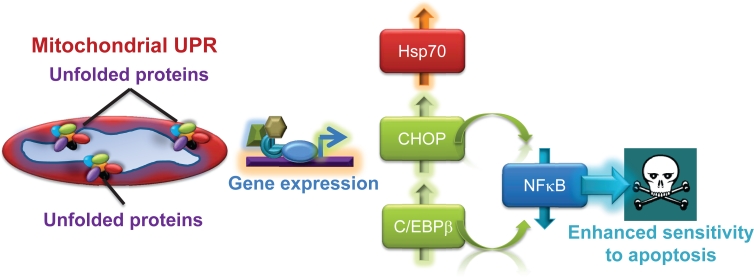
Exploitation of a mitochondrial UPR for cancer therapy Mitochondrial dysfunction associated with proteotoxic stress results in the upregulation of stress response transcription factors, CHOP and C/EBPβ, which in turn repress NFκB activity, sensitizing tumor cells to apoptosis-inducing agents.

Second, mitochondria isolated from glioblastoma cells treated with low concentrations of Gamitrinib showed accumulation of unfolded, i.e. insoluble proteins, deregulated expression of “sensor” proteins of organelle damage, and a “stress response” gene signature characterized by upregulation of chaperones, for instance Hsp70, and transcription factors, CCAAT-enhancer binding protein, C/EBPβ, and its dimerization partner, CHOP [6] (Fig. 1). Together, these are all markers of a mitochondrial unfolded protein response (UPR) [12-14], and genetic knockdown of the targets of Gamitrinib in mitochondria, TRAP-1 or CypD, reproduced this pathway independently of pharmacologic inhibition. Intriguingly, one of the hallmarks of this mitochondrial UPR was a complete ablation of NFκB transcriptional activity, either constitutive or in response to TNFα, with concomitant loss of multiple downstream NFκB-inducible genes [6] (Fig. 2). The suppression of NFκB activity under these conditions was mediated by upregulated CHOP and C/EBPβ, but was not part of a general inhibitory effect on gene expression, and was specific for the mitochondrial chaperones, as a non-subcellularly-targeted Hsp90 inhibitor, 17-AAG, had no effect [6] (Fig. 2).

There is a wealth of literature pointing to a pivotal role of NFκB in tumor progression. In addition to modulating an inflammatory tumor microenvironment, NFκB functions as a potent survival mechanism in tumors, upregulating the expression of multiple anti-apoptotic genes, including cFLIP, a negative regulator of death receptor-induced apoptosis [15]. This response has important implications in the clinic, because high NFκB activity is typically associated with treatment resistance and worse outcome in cancer patients. Together, this raised a testable hypothesis, whether tumor cells undergoing mitochondrial UPR and concomitant loss of NFκB-activity (Fig. 2), were now re-sensitized to apoptosis-based therapy. For these experiments, we used TNF Receptor Apoptosis-Inducing Ligand (TRAIL), a potent pro-apoptotic death receptor ligand currently in clinical trials, but whose efficacy is often reduced or abolished by NFκB. Instead, a combination regimen of low, i.e. non-cytotoxic concentrations of Gamitrinib plus TRAIL dramatically killed tumor cells normally resistant to TRAIL, and potently inhibited intracranial glioblastoma growth in mice with no detectable toxicity [6].

## UNANSWERED QUESTIONS AND FUTURE DIRECTIONS

Overall, these data [6] open fresh perspectives into how tumor cells cope with compartmentalized, i.e. mitochondrial proteotoxic stress, and mount a multipartite response that attempts to preserve cell survival and restore homeostasis (Fig. 1). Much of our knowledge about organelle-initiated UPR comes from studies of ER damage [16]. In contrast, the nature of a mitochondrial UPR has remained largely elusive, complicated by the unique architecture of the organelle, its production of protein-altering reactive oxygen species, and the dual role of matrix proteases and chaperones in preprotein import and (re)folding [17]. Although we knew that artificially-induced mitochondrial damage could lead to an UPR [12-14], what we did not know were the physiologic requirements of this pathway, and its broader implications for cellular homeostasis. The recent results [6] now suggest that a mitochondrial UPR may be a relatively common occurrence in tumors that undergo non-fatal mitochondrial damage, and identify mitochondrial Hsp chaperones as physiologic mediators of this response. It is also clear that not all mitochondrial damage induces an UPR, because organelle outer membrane depolarization, or induction of apoptosis had no effect [6]. As to the functional question whether this response is friend or foe, it seems reasonable to speculate that the combined activation of autophagy and stress response genes (Fig. 1) provide a powerful compensatory mechanism to eliminate the damaged organelles, while globally elevating the cell's buffering and survival threshold, respectively [17]. We still do not know how this combined adaptive and cytoprotective mechanism becomes so dramatically exploited in cancer [5]. A possible model is that tumor cells hijack this response from a normal counterpart, and, in this case, neurons are interesting candidates for potential exploitation, as these cells rely on cytoprotection from mitochondrial Hsps to antagonize CypD-dependent apoptosis [18], and preserve cell survival [19]. Although activation of the mitochondrial UPR [6] may worsen disease outcome, similar to how other stress response mechanisms facilitate tumor progression [20], the profound suppression of NFκB seen under these conditions [6], may open concrete opportunities to re-sensitize resistant tumors to apoptosis-inducing agents, a critical goal of modern cancer therapy [21] (Fig. 2).

Clearly, important questions remain to be answered. First, it is unclear how inhibition of mitochondrial Hsp90s, and the ensuing organelle proteotoxic stress (Fig. 1), couple to nuclear activation of gene expression. Examples from other organelle-initiated stress response pathways may help pointing to future experiments [22], and second messengers potentially generated by changes in mitochondrial bioenergetics, deregulated Ca^2+^ homeostasis or intramitochondrial proteolysis may be explored as effectors of this response [23]. A second important question is whether the signal generated through the mitochondrial UPR requires amplification by the ER stress-sensing machinery to fully activate gene expression. The concept of inter-organelle communication is emerging more and more often in disparate experimental systems, and, in particular, ER and mitochondria share extensive areas of physical contact, populated by an elaborate stress-sensing machinery that couples to gene expression. And, finally, there is the question of how this mitochondrial UPR actually represses NFκB activity, when other organelle signaling pathways are instead typically associated with its induction [16, 24]. Here, it will be of interest to test a link between the transcriptional effectors of the mitochondrial UPR and the plethora of NFκB activators or inhibitors that control the balance between induction and silencing of this pathway, especially in tumor cells.

For now, the recent results [6] have uncovered a new layer of complexity for how tumor cells adjust to their aberrantly higher biosynthetic needs, and control the mitochondrial protein folding environment against the risk of proteotoxic stress (Fig. 1). The recent proof-of-concept studies [6] reinvigorate the concept of Hsp90 chaperones as important nodal targets for cancer therapeutics, but also underscore that only targeting their inhibitors to specialized subcellular microenvironments, for instance mitochondria [7], can achieve the kind of anticancer efficacy that bodes well for human testing. And, finally, it is now possible to envision molecularly-grounded synergistic anticancer therapies, exemplified by the combination of Gamitrinib plus TRAIL, which turn the table on a general adaptive and survival mechanism (Fig. 2) to deliver promising activity in models of notoriously recalcitrant human tumors.
